# Antimicrobial Synergy of Silver-Platinum Nanohybrids With Antibiotics

**DOI:** 10.3389/fmicb.2020.610968

**Published:** 2021-02-01

**Authors:** Bansi Ranpariya, Gayatri Salunke, Srikanta Karmakar, Kaushik Babiya, Santosh Sutar, Narendra Kadoo, Pathik Kumbhakar, Sougata Ghosh

**Affiliations:** ^1^Department of Microbiology, School of Science, RK University, Rajkot, India; ^2^Biochemical Sciences Division, CSIR-National Chemical Laboratory, Pune, India; ^3^Academy of Scientific and Innovative Research, Ghaziabad, India; ^4^Nanoscience Laboratory, Department of Physics, National Institute of Technology Durgapur, Durgapur, India; ^5^Yashwantrao Chavan School of Rural Development, Shivaji University, Kolhapur, India

**Keywords:** biogenic synthesis, silver-platinum nanohybrids, characterization, antimicrobial synergy, antibiofilm

## Abstract

Various bacterial pathogens are responsible for nosocomial infections resulting in critical pathophysiological conditions, mortality, and morbidity. Most of the bacterial infections are associated with biofilm formation, which is resistant to the available antimicrobial drugs. As a result, novel bactericidal agents need to be fabricated, which can effectively combat the biofilm-associated bacterial infections. Herein, for the first time we report the antimicrobial and antibiofilm properties of silver-platinum nanohybrids (AgPtNHs), silver nanoparticles (AgNPs), and platinum nanoparticles (PtNPs) against *Escherichia coli, Pseudomonas aeruginosa*, and *Staphylococcus aureus*. The AgPtNHs were synthesized by a green route using *Dioscorea bulbifera* tuber extract at 100°C for 5 h. The AgPtNHs ranged in size from 20 to 80 nm, with an average of ∼59 nm. AgNPs, PtNPs, and AgPtNHs showed a zeta potential of −14.46, −1.09, and −11.39 mV, respectively. High antimicrobial activity was observed against *P. aeruginosa* and *S. aureus* and AgPtNHs exhibited potent antimicrobial synergy in combination with antibiotics such as streptomycin, rifampicin, chloramphenicol, novobiocin, and ampicillin up to variable degrees. Interestingly, AgPtNHs could inhibit bacterial biofilm formation significantly. Hence, co-administration of AgPtNHs and antibiotics may serve as a powerful strategy to treat bacterial infections.

## Introduction

Recently, nanobiotechnology has got wide attention due to multiple applications in electronics, catalysis, textiles, food industries, and therapeutics. Among various nanoparticles, silver nanoparticles (AgNPs) are used in biosensing, biomedical imaging, and drug delivery (Tarannum et al., 2019). Further, AgNPs are also used for designing antimicrobial surfaces, cosmetics, paints, and plastics. Because of their bactericidal and fungicidal properties, AgNPs are also used for the fabrication of wound dressings (Wilkinson et al., 2011; Robkhob et al., 2020). Similarly, platinum nanoparticles (PtNPs) are also known for their excellent antimicrobial activity and ability to inhibit the growth of unwanted and harmful bacteria (Tahir et al., 2017).

Bacterial biofilms are complex communities where the bacterial cells adhere to the surface and each other being embedded in a protective exopolymeric substance. Induction of multidrug resistance in the biofilm associated cells is attributed to the enhanced cell-to-cell communication (quorum sensing), and notable exchange of genetic material by horizontal gene transfer. It is speculated that the bacteria growing in biofilms are often thousands of times more tolerant to antimicrobial treatment than their planktonic counterparts (Verderosa et al., 2019). Thus, it is very difficult to treat nosocomial infections associated with pathogenic biofilm on implants, catheters, stents, heart valves, and pacemakers that pose a potential health risk (Francolini and Donelli, 2010). Clinically significant pathogens like *Pseudomonas aeruginosa* (Gellatly and Hancock, 2013), *Escherichia coli* (Beloin et al., 2008), and *Staphylococcus aureus* (Gordon and Lowy, 2008) have exhibited biofilm formation as predominant virulence mechanism. Biofilm associated diseases like vaginitis (Machado et al., 2016), otitis (Post, 2001), gingivitis (Vieira Colombo et al., 2016), conjunctivitis (Behlau and Gilmore, 2008), urethritis (Delcaru et al., 2016), and colitis (von Rosenvinge et al., 2013) are challenging to treat. *P. aeruginosa* biofilms in the lungs of cystic fibrosis patients is a serious medical concern which is known to cause acute and chronic lung infections resulting in significant morbidity and mortality (Wagner and Iglewski, 2008). Chronic wound infections caused by *P. aeruginosa* and *S. aureus* (Omar et al., 2017), are reported to be responsible for over 80% of the 100,000 limb amputations carried out every year in diabetic patients (James et al., 2008). Similarly, Moazzezy et al. (2020) reported *E. coli* to be highly heterogeneous group of biofilm forming uropathogens causing urinary tract infection. The bacterial biofilms is challenging to treat with available antibiotics because the drug cannot penetrate in the deeper parts of the biofilm. Moreover, several other mechanisms existing in the biofilm forming bacteria like enzymatic degradation of the antibiotics, efflux pumps and alteration of the target site by mutations render the drug ineffective. Hence, there is a need to develop novel antimicrobial agents that can significantly inhibit biofilm formation. Nanoparticles with efficient bactericidal effects and antibiofilm effects have come up as potential alternative and complementary agents against biofilm associated microbial infections.

Various physical and chemical methods for synthesizing nanoparticles, have been reported, which include chemical reduction, template method, electrochemical, or ultrasonic-assisted reduction, photoinduced or photocatalytic reduction, microwave-assisted synthesis, irradiation reduction, microemulsion, and biochemical reduction. These methods involve toxic chemicals and hazardous conditions and significantly compromise the biocompatibility of the resulting nanoparticles for the biomedical applications (Jamkhande et al., 2019). Hence, there is an urgent need to develop green and environmentally benign route which will help to synthesize nanoparticles with broad-spectrum therapeutic potential.

Several bacteria, fungi, algae, and medicinal plants have been employed to synthesize nanoparticles of gold, silver, copper, platinum, and palladium, etc. (Iravani, 2011). Synthesis of nanoparticles using microbes requires a tedious culturing process, optimization, aseptic condition, and downstream processing. Whereas, medicinal plants with numerous phytochemical diversities such as terpenes, polyphenols, flavonoids, alkaloids, coumarin, and saponins have served as attractive materials for both reduction of metal ions to their corresponding nanoparticles and their stabilization (Singh et al., 2016). Several plants such as *Ocimum tenuiflorum, Solanum trilobatum, Syzygium cumini, Centella asiatica*, *Citrus sinensis, Carica papaya, Citrus limon, Desmodium triflorum, and Euphorbia hirta*, etc. have been reported to synthesize metal nanoparticles with exotic shapes and sizes with significant biomedical applications (Iravani, 2011; Logeswari et al., 2015). Microbial interaction with the biogenic nanoscale metals is noteworthy as significant bactericidal efficacy is exhibited by metal nanoparticles in compared to their bulk counterparts. Further, synergistic antimicrobial action with multimetal complexes can be of utmost significance as they might induce higher oxidative stress thereby efficiently killing the microbes.

Herein, we report the enhanced bactericidal activity of the biogenic silver-platinum bimetallic nanohybrids (AgPtNHs) which was synthesized using *Dioscorea bulbifera* tuber extract. Further, the antimicrobial synergy with various antibiotics was also evaluated. The effect of the AgPtNHs on the biofilm-forming activity of the microbes was also examined and the morphological alterations of the bacterial biofilms on treatment with the AgPtNHs have been studied by using scanning electron microscopic and atomic force microscopic analyses.

## Materials and Methods

### Synthesis of AgPtNHs

*Dioscorea bulbifera* tuber extract (DBTE) was prepared as per our earlier protocol (Ghosh et al., 2012). The synthesis of AgPtNHs was achieved by the addition of 5 mL of DBTE in 95 mL of an aqueous solution containing 10^–3^ M of both H_2_PtCl_6_.6H_2_O and AgNO_3_ followed by incubation at 100°C for 5 h. Synthesis of only AgNPs was achieved by reacting 5 mL of freshly prepared DBTE with 95 mL of 10^–3^ M aqueous AgNO_3_ solution at 40°C for 5 h. PtNPs were synthesized due to the reduction of PtCl_6_^2–^ ions on the addition of 5 mL of DBTE to 95 mL of 10^–3^ M aqueous solution of H_2_PtCl_6_.6H_2_O which was incubated at 100°C for 5 h. The synthesis of the material was confirmed by recording the UV-visible spectrum of the solution after 5 h on a spectrophotometer (SpectraMax M5, Molecular Devices Corporation, Sunnyvale, CA, United States) operating at a resolution of 1 nm.

### Characterization

After completing the synthesis, preliminary confirmations of biosynthesized AgNPs, PtNPs, and AgPtNHs were carried out through visual observation of color change. The bioreduced nanoparticles were further characterized by using several standard techniques, such as UV-vis spectroscopy, transmission electron microscopy (TEM), energy dispersive spectra (EDS), and dynamic light scattering (DLS).

### Fourier-Transform Infrared Spectrophotometry

Fourier-Transform Infrared Spectrophotometry (FTIR) was employed to understand the underlying mechanism of the synthesis of the nanoparticles using DBTE. In this method, DBTE after and before synthesis of AgPtNHs was subjected to Fourier-transform infrared (FTIR, IRAffinity-1, Shimadzu Corporation, Tokyo, Japan) spectroscopy measurement using the potassium bromide (KBr) pellet technique in diffuse reflection mode at a resolution of 4 cm^–1^. An infrared source of wavelength lying within 500–4,000 cm^–1^ was used.

### Antimicrobial Activity

The effects of AgNPs, PtNPs, and AgPtNHs were evaluated against *E. coli*, *P. aeruginosa*, and *S. aureus*, on Mueller Hinton Agar (MHA) plates using well diffusion assay. Overnight grown cultures of the test organisms (OD_600_ = 0.05) were spread plated on MHA plates and wells were made on the surface with the help of a sterile cork borer of diameter 5 mm. 30 μL of nanoparticles suspension (100 μg/mL) was added in the wells, followed by incubation at 37°C for 18 h, the zone of inhibition was measured (Karmakar et al., 2020).

### Antimicrobial Synergy

Agar well diffusion technique was used for evaluating the antimicrobial synergy of the nanoparticles in the presence of antibiotics. The antibiotics used were streptomycin, rifampicin, chloramphenicol, novobiocin, and ampicillin. The test pathogens were inoculated in sterile Mueller Hinton Broth (MHB) and incubated in a shaker for overnight at 37°C. 100 μL of the overnight grown culture was spread uniformly on MHA plates. Wells were made on the agar surface with the help of a sterile cork borer with 6 mm diameter and 30 μL of the nanoparticles, a mixture of nanoparticles and antibiotics, and only antibiotics (100 μg/mL) were added. The plates were then incubated at 37°C for 18 h and observed for a zone of inhibition around the well. The diameters of the zone of inhibition were measured and the degree of antimicrobial synergy was evaluated. All experiments were performed in triplicates.

### Antibiofilm Activity

The biofilm inhibitory potential of the nanoparticles was evaluated by using the gentian violet staining method (Ghosh et al., 2015a). In brief, 5 μL of overnight grown bacterial cultures (OD adjusted to 0.05 at 600 nm) of *E. coli*, *P. aeruginosa*, and *S. aureus* were incubated in the absence and in the presence of nanoparticles (at a final concentration of 10 μg/well) supplemented in MHB in 96 well microtitre plates. The microtitre plates were then incubated for 48 h at 37°C under static conditions. Thereafter, nonadherent cells were removed by aspiration and the wells with biofilm were washed thrice with sterile phosphate-buffered saline (PBS). Then 0.1% gentian violet was added in each well and incubated for 10 min at room temperature, and the excess stain was removed by repeated washing with water. The wells were then dried in a laminar air flow and 200 μL of absolute ethanol was added to each well and further shaken at 1,020 rpm for 10 s. The value of absorbance at 570 nm was recorded in a multiplate reader. Biofilm indices were calculated after normalizing with appropriate controls. All biofilm assays were repeated thrice.

### Biofilm Visualization by Atomic Force Microscopy

The biofilm inhibition was carried out on sterile grease-free glass coverslips as per the procedure mentioned above with a final volume of 2 mL in six well plates. The biofilms of *E. coli, P. aeruginosa*, and *S. aureus* were allowed to form on glass slides incubated for 48 h in the presence of nanoparticles (at a final concentration of 10 μg/mL). The glass coverslips were washed with sterile PBS followed by fixation with glutaraldehyde and sequential dehydration with ethyl alcohol and then dried in a vacuum. Morphological features of the untreated and treated biofilms on the glass surfaces were analyzed by using atomic force microscopic (AFM) imaging. Atomic force measurements were carried out using a multimode scanning probe microscope (Model number MMAFMLN, VeecoMetrology, Santa Barbara, CA, United States) equipped with a Nanoscope VI controller. AFM micrographs were generated in tapping mode with the probe, tap190 (Budget SensorsAFM tips, Bulgaria).

### Statistical Analysis

All values were expressed as mean ± standard error of mean (S.E.M.), *n* = 3, and statistical significance was determined by analysis of variance (ANOVA two factor) with ^∗^*P* < 0.05.

## Results

### Synthesis and Characterization of AgPtNHs

**FIGURE 1:**
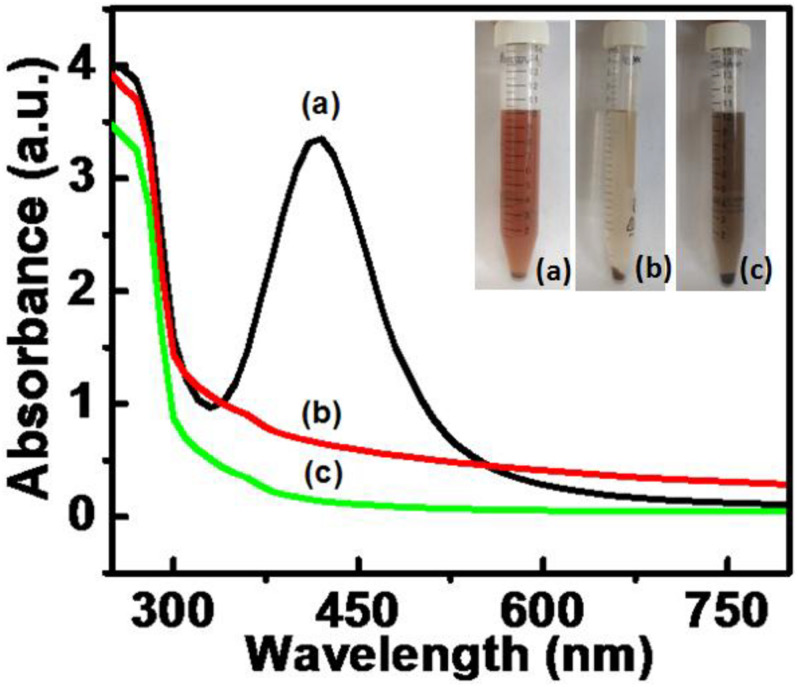
UV-visible spectra for nanoparticles synthesized by DBTE with insets representing the visible color change on complete synthesis after 5 h. **(a)** AgNPs; **(b)** AgPtNHs; and **(c)** PtNPs.

The syntheses of AgNPs, PtNPs, and AgPtNHs were completed in 5 h. The digital images of the samples taken after the synthesis is presented in the inset of Figure 1 and the appearance of brown color confirmed the formation of the nanoparticles. AgNPs with light brown color were well dispersed in the colloidal solution while PtNPs settled down like a loose mass with blackish-brown color. On the other hand, AgPtNHs formed relatively well dispersed dark colloidal suspension. The UV-Vis. absorption spectra of the samples were measured, as shown in Figure 1. The AgNPs exhibited a prominent peak at 420 nm in the UV-visible spectra while PtNPs and AgPtNHs showed a formless peak.

**FIGURE 2:**
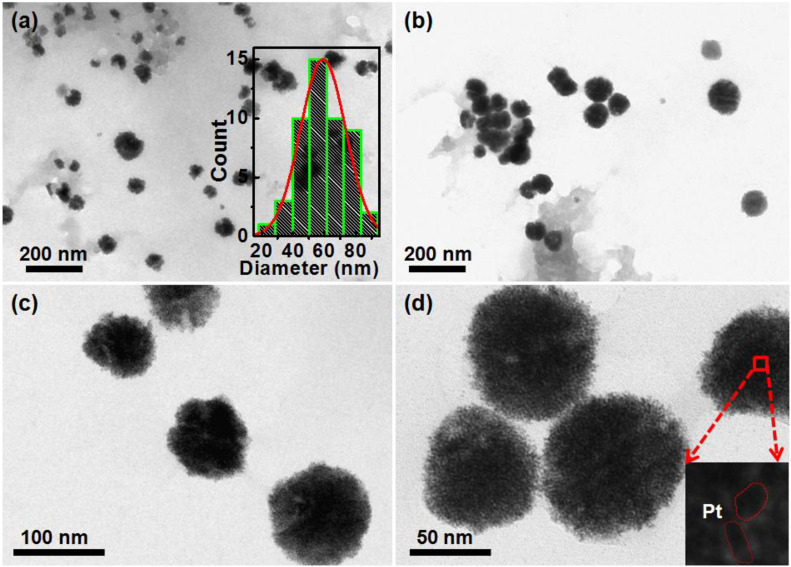
TEM image of AgPtNHs synthesized by DBTE. **(a)** Images showing nanoclusters and inset showing histogram of particle size distribution; **(b)** Discrete AgPtNHs; **(c)** Magnified images of AgPtNHs; and **(d)** Granular nanoparticles assembling to form larger nanoclusters.

Further, the morphologies of the synthesized AgPtNHs, were studied using TEM. As evident from the TEM micrographs in Figure 2, very small independent nanoparticles of AgPtNHs with a size of ∼2 nm assembled to form nanoclusters with the overall size distribution from 20 to 80 nm with the average being 59 nm. The AgPtNHs were stable and discrete and formed spherical shaped nanoclusters. Also, the nanoparticles were seen to be stabilized by the biological components of the plant extract. The inset of Figure 2d shows that the granular AgPtNHs are assembling to form larger nanoclusters.

**FIGURE 3:**
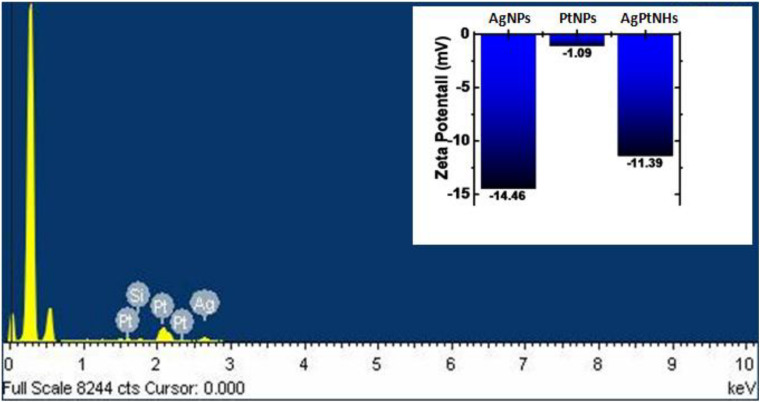
Representative spot EDS profile confirming the presence of Ag and Pt in the AgPtNHs synthesized by DBTE. Inset represents zeta potential values of the biogenic nanoparticles.

The EDS measurement was done to find out the existence of Ag and Pt in the AgPtNHs, as shown in Figure 3. The spectra confirmed the presence of both elemental Ag and Pt in the AgPtNHs. Further, zeta potential measurements were carried out to evaluate the stability of the nanoparticles because the antimicrobial activity is a function of stability of nanoparticle. Zeta values, as shown in the inset of Figure 3, further rationalize the observation where AgNPs showed more negative value (−14.46 mV) followed by AgPtNHs (−11.39 mV) and PtNPs (−1.09 mV).

### FTIR Analysis

**FIGURE 4:**
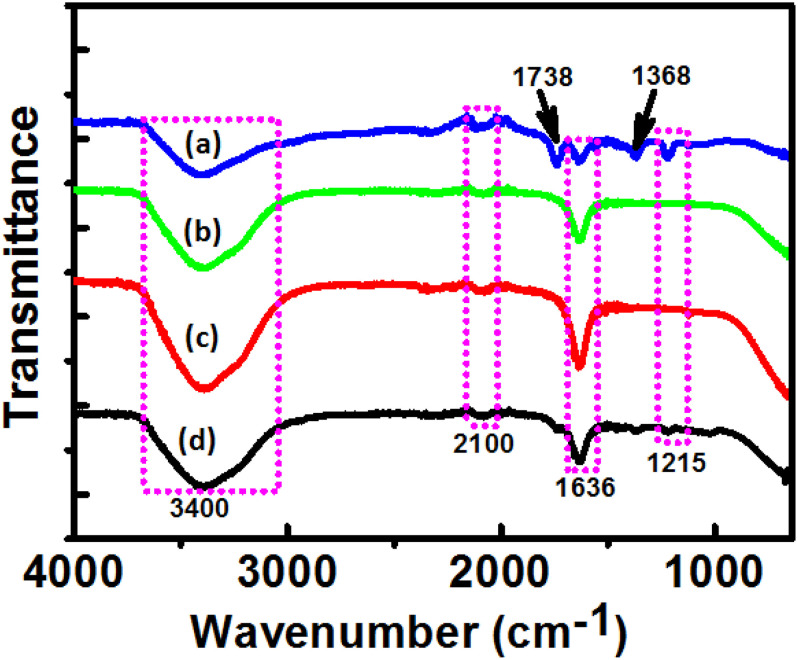
Fourier transform infrared absorption spectra of dried *Dioscorea bulbifera* tuber extract (DBTE) after complete bioreduction of **(a)** AgNPs; **(b)** PtNPs; **(c)** AgPtNHs; and **(d)** before bioreduction.

Fourier-transform infrared (FTIR) spectra of DBTE before and after synthesis of AgNPs, PtNPs, and AgPtNHs is presented in Figure 4. Some typical peaks observed at 3,400, 2,100, 1,636, and 1,215 cm^–1^ were present in all the spectra. Those peaks might have originated from the different vibration bands (i.e., flavonoids, terpenoids, phenanthrenes, amino acids, proteins, and glycosides) present within the DBTE. The strong peak at ∼3,400 cm^–1^ is a characteristic of the hydroxyl group in polyphenolic compounds present in the plant extract. The other bands at 2,100, 1,636, and 1,212 cm^–1^ are assigned to C≡C stretching of the alkyne, C=C groups, or conjugated C–C with a benzene ring phenolic groups (Ghosh et al., 2011, 2012). There were some additional peaks which appeared after synthesis that included 1,738 and 1,368 cm^–1^, which might have originated from C=O carbonyl stretch from carboxylic acid and C–N stretching vibration of aromatic ring, respectively (Song et al., 2010; Ghosh et al., 2012). The sharpness of the peak representing the O–H bond was reduced in the FTIR spectrum of DBTE after synthesis of the nanoparticles, which confirmed the bioreduction efficiency of DBTE. Therefore, the FTIR results demonstrated that the DBTE could perform dual functions of reduction and stabilization of AgNPs, PtNPs, and AgPtNHs. The overall observation confirmed that the presence of complex compounds in DBTE could bind to nanoparticles either through free amine groups via electrostatic attraction of negatively charged carboxylate groups and therefore stabilized the phytogenic nanoparticles (Vigneshwaran et al., 2007).

### Antimicrobial Activity

**FIGURE 5:**
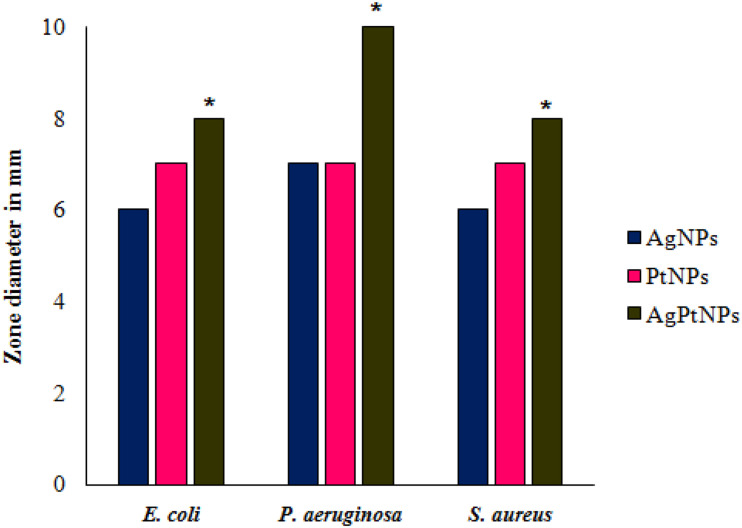
Antimicrobial activity of biogenic nanoparticles synthesized by DBTE. *P < 0.05; the mean difference in the antimicrobial activity is significant in AgPtNHs at the 0.05 level by two factor ANOVA.

The biogenic nanoparticles showed variable degrees of antimicrobial activity against the three test pathogens as represented in Figure 5 in terms of zone of inhibition. AgNPs showed the highest activity against *P. aeruginosa* while identical inhibition was observed against *E. coli*, and *S. aureus*. PtNPs also showed a similar level of inhibition against all bacteria. Interestingly, the inhibitory activity of AgPtNHs was more against all test pathogens in compared to individual AgNPs or PtNPs. AgPtNHs showed the highest inhibitory zone of 10 mm against *P. aeruginosa* followed by *E. coli* (8 mm), and *S. aureus* (8 mm).

### Antimicrobial Synergy

To study the antimicrobial synergy, the activities of the individual antibiotics and in combination with the nanoparticles were evaluated and the fold increase in terms of the zone of inhibition was determined. Table 1 shows the synergistic antimicrobial activity of various antibiotics in combination with AgNPs. The activity of streptomycin was found to get enhanced 8 folds against *E. coli* when supplemented with AgNPs. Rifampicin exhibited the highest antimicrobial synergy in combination with AgNPs against *S. aureus* (15 folds) followed by intermediate activity against *E. coli* (4.64 folds). Chloramphenicol showed intermediate synergy in presence of AgNPs against both *E. coli* and *S. aureus.* Novobiocin exhibited high synergistic inhibition of *S. aureus* (15 folds) followed by *E. coli*. Interestingly, ampicillin exhibited selectivity toward Gram-positive bacteria during synergistic activity in combination with AgNPs. *S. aureus* was inhibited up to 12.14 folds by the combination of ampicillin and AgNPs.

**TABLE 1:** Zone of inhibition (mm) of different antibiotics against bacteria in the absence and in the presence of AgNPs (30 μg/well).

Antibiotics	E. coli*			P. aeruginosa*			S. aureus*		
			
	A	B	C	A	B	C	A	B	C
Streptomycin	7	21	8.00	8	14	2.06	9	15	1.78
Rifampicin	8	19	4.64	8	9	0.27	8	32	15.00
Chloramphenicol	8	22	6.56	7	9	0.65	7	20	7.16
Novobiocin	7	22	8.88	8	10	0.56	6	24	15.00
Ampicillin	8	14	2.06	7	8	0.31	8	29	12.14

All the experiments were performed in triplicate, and standard deviations were negligible. Fold increases (C) for different antibiotics against three bacterial pathogens were calculated as (B^2^ – A^2^)/A^2^, where A, B are the inhibition zones in mm for antibiotic only and antibiotic in combination with AgNPs, respectively. In the absence of bacterial growth inhibition zones, the well diameters (6 mm) were used to calculate the fold increase (C). *P < 0.05; the mean difference in the synergy is significant among the bacteria at the 0.05 level by two factor ANOVA.

The antimicrobial synergy of antibiotics in combination with PtNPs showed the selectivity and variability as observed in Table 2. Supplementation with PtNPs significantly enhanced the inhibitory activity of streptomycin selectively against Gram-negative bacteria *E. coli* (10.76 folds) and *P. aeruginosa* (8 folds). In the presence of PtNPs antimicrobial activity of rifampicin was increased notably against Gram-positive bacteria, *S. aureus* (16.02 folds). Likewise, increment in the activity of chloramphenicol in the presence of PtNPs was notable against Gram-positive pathogen *S. aureus* (8 folds). The highest antimicrobial synergy of novobiocin with PtNPs was seen against *S. aureus* (16.36 folds), followed by *E. coli* (10.76 folds) while intermediate and low synergy was evident against *P. aeruginosa.* As observed in Table 3, antimicrobial activity of rifampicin and novobiocin increased significantly up to 15 and 13.69 folds, respectively, in combination with AgPtNHs against *S. aureus*.

**TABLE 2:** Zone of inhibition (mm) of different antibiotics against bacteria in the absence and in the presence of PtNPs (30 μg/well).

Antibiotics	E. coli*			P. aeruginosa*			S. aureus*		
			
	A	B	C	A	B	C	A	B	C
Streptomycin	7	24	10.76	8	24	8.00	9	13	1.09
Rifampicin	8	13	1.64	8	12	1.25	8	33	16.02
Chloramphenicol	8	19	4.64	7	11	1.47	7	21	8.00
Novobiocin	7	24	10.76	8	14	2.06	6	25	16.36
Ampicillin	8	18	4.06	7	12	1.94	8	15	2.52

All the experiments were performed in triplicate, and standard deviations were negligible. Fold increases (C) for different antibiotics against three bacterial pathogens were calculated as (B^2^ – A^2^)/A^2^, where A, B are the inhibition zones in mm for antibiotic only and antibiotic in combination with PtNPs, respectively. In the absence of bacterial growth inhibition zones, the well diameters (6 mm) were used to calculate the fold increase (C). *P < 0.05; the mean difference in the synergy is significant among the bacteria at the 0.05 level by two factor ANOVA.

**TABLE 3:** Zone of inhibition (mm) of different antibiotics against bacteria in the absence and in the presence of AgPtNHs (30 μg/well).

Antibiotics	E. coli*			P. aeruginosa*			S. aureus*		
			
	A	B	C	A	B	C	A	B	C
Streptomycin	7	20	7.16	8	12	1.25	9	15	1.78
Rifampicin	8	15	2.52	8	11	0.89	8	32	15.00
Chloramphenicol	8	20	5.25	7	10	1.04	7	18	5.61
Novobiocin	7	23	9.80	8	8	0.00	6	23	13.69
Ampicillin	8	13	1.64	7	13	2.45	8	15	2.52

All the experiments were performed in triplicate, and standard deviations were negligible. Fold increases (C) for different antibiotics against three bacterial pathogens were calculated as (B^2^ – A^2^)/A^2^, where A, B are the inhibition zones in mm for antibiotic only and antibiotic in combination with AgPtNHs, respectively. In the absence of bacterial growth inhibition zones, the well diameters (6 mm) were used to calculate the fold increase (C). *P < 0.05; the mean difference in the synergy is significant among the bacteria at the 0.05 level by two factor ANOVA.

### Antibiofilm Activity

**FIGURE 6:**
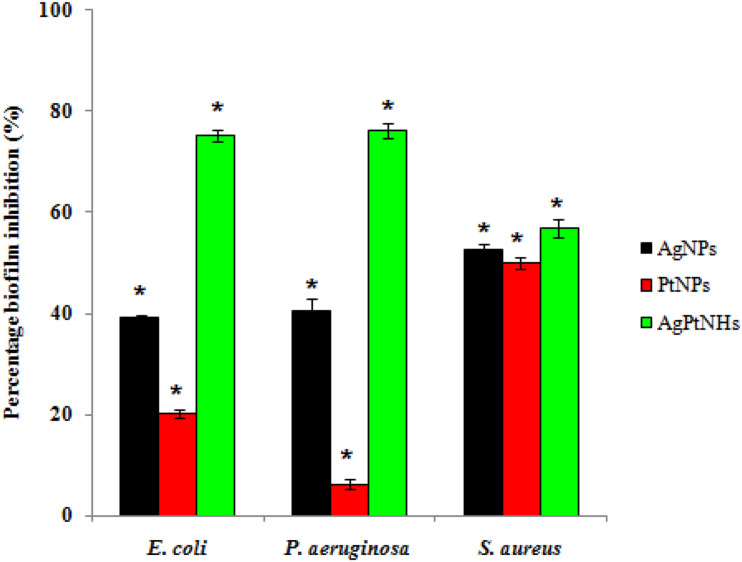
Biofilm inhibition by biogenic nanoparticles synthesized by DBTE. The data is indicated as the mean ± SEM; (*n* = 3).*P < 0.05; the mean difference in the antibiofilm activity is significant among the nanoparticles at the 0.05 level by two factor ANOVA.

The effect of phytogenic nanoparticles on the biofilm-forming ability of the bacterial pathogens was checked which revealed variability in the degree of inhibition as represented in Figure 6. A high degree of biofilm inhibition was observed on treatment with AgPtNHs while treatment with PtNPs showed a comparatively lower biofilm inhibition. *E. coli* and *P. aeruginosa* showed almost identical levels of biofilm inhibition up to 75.16 ± 1.02% and 76.18 ± 1.42% in the presence of AgPtNHs. Pure AgNPs, on the other hand showed 39.11 ± 0.52% and 40.49 ± 2.47% biofilm inhibition against *E. coli* and *P. aeruginosa*, respectively, while inhibition by PtNPs was lower. Inhibition of *S. aureus* biofilm with AgPtNHs (56.7 ± 1.81%) was more as compared to both AgNPs (52.72 ± 0.84%) and PtNPs (49.98 ± 1.23%).

### Atomic Force Microscopy

**FIGURE 7:**
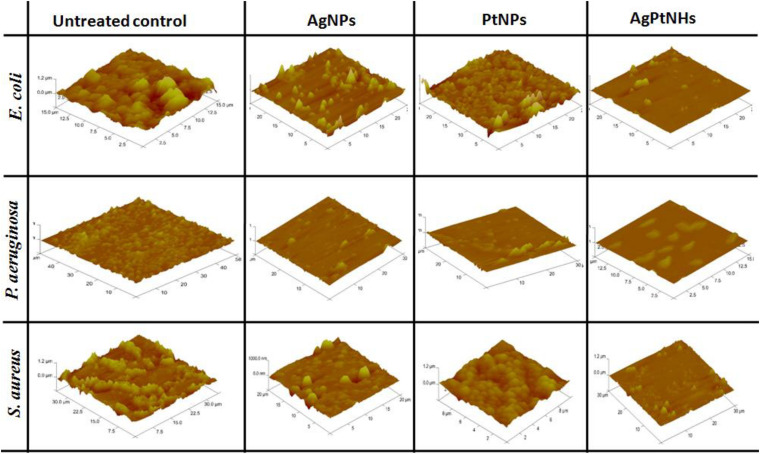
Atomic force microscopic images of untreated and treated biofilms.

The effect of biogenic nanoparticles on bacterial biofilms was confirmed by using AFM image analysis. Figure 7 depicts the prominent differences between the architecture of untreated and treated bacterial biofilms on the glass surface. Untreated biofilms showed a packed lawn of bacteria without exposing the glass surface underneath. Treatment with AgNPs compromised the cell adhering capability in *E. coli* resulting in the interrupted biofilm while AgPtNHs showed high biofilm elimination. Untreated *P. aeruginosa* biofilms showed uniformly embedded bacterial cells in the polymeric matrix that was significantly reduced on treatment with AgNPs, PtNPs, and AgPtNHs.

Atomic force microscopy observations could be strongly correlated with the antibiofilm activity where overall biofilm inhibition against *S. aureus* was lower in compared to other bacteria. In spite of treatment with AgNPs and PtNPs, the glass surfaces were covered with *S. aureus* biofilms which reduced substantially on treatment with AgPtNHs.

## Discussion

Biological synthesis of nanoparticles is widely preferred due to non-involvement of harmful toxic chemicals which otherwise make the resulting nanoparticles non-biocompatible and hazardous during therapeutic application. Biogenic routes of synthesis are rapid, efficient and economical as the metabolites in the extracts generally act as potential reducing as well as stabilizing agents (Garg et al., 2020).

*Dioscorea bulbifera*, commonly known as air potato, has numerous medical applications owing to its inherent antibacterial, antifungal, plasmid curing, antidiabetic, antioxidant, and anticancer properties. Traditionally it has been used as a purgative, aphrodisiac, anthelmintic, rejuvenating tonic, diuretic, deflatulent and has been widely used for ameliorating scrofula, hemorrhoids, hematological disorders, diabetic disorders, polyurea, worm infestations, and skin diseases (Ghosh et al., 2015b; Kundu et al., 2020).

The therapeutic activity of the medicinal plants is attributed to the rich phytochemistry. Several medicinal plants such as *Callicarpa maingayi, Cissus quadrangularis, Tribulus terrestris, Centella asiatica, Murraya koenigii, Alternanthera sessilis, Artemisia nilagirica*, and many more are thus explored for the synthesis of various metal nanoparticles (Kuppusamy et al., 2016). Notably, the earlier reports are mostly on AgNPs, AuNPs, CuNPs, and other individual nanoparticles. A very few reports exist on bi-metallic nanoparticles synthesized by using medicinal plants. Zhan et al. (2011) reported Au–Pd bimetallic nanoparticles with approximately 7 nm size and well defined spherical shape using *Cacumen platycladi* leaf extract in an aqueous environment. The synthesis was complete after incubation for 2 h under vigorous stirring. Likewise, Salunke et al. (2014) showed that aqueous root extract of *P. zeylanica* (PZRE) rendered hexagonal blunt-ended AgAuNPs with a size of 90 nm apart from spherical AgNPs (60 nm), and triangular AuNPs (20–30). The resulting nanoparticles showed efficient antimicrobial and antibiofilm activities. In another study, ginger rhizome powder (GP) was used to fabricate three different bimetallic catalysts namely copper-silver (Cu-Ag/GP), copper-nickel (Cu-Ni/GP), and nickel-silver (Ni-Ag/GP) complexes employing a robust adsorption method for applications in catalytic dye degradation (Ismail et al., 2018). However, this is the first report on the synthesis of AgPtNHs using *D. bulbifera* as reducing and stabilizing agent which was completed within 5 h time at 100°C temperature. This can be the potential green route for the rational fabrication of various other nanohybrids.

The appearance of blackish brown color at 100°C indicated that the completion of both AgPtNHs and PtNPs syntheses were facilitated at higher temperature. A similar observation was reported during synthesis of PtNPs using *Diopyros kaki* leaf extract where increasing the reaction temperature up to 95°C resulted in almost 100% conversion of platinum ions to PtNPs (Song et al., 2010). Evidences of featureless peak were also found during synthesis of platinum palladium bimetallic nanoparticles (PtPdNPs) in our earlier report (Ghosh et al., 2015c). Unlike core–shell bimetallic nanoparticles, which display two bands in UV-visible absorption spectra, the featureless spectra AgPtNHs indicated the possible formation of a nanoalloy (Elemike et al., 2019; Unuofin et al., 2020).

The exotic shape of the AgPtNHs was found to be very small nanospheres aggregated as spherical nanoclusters with an average size of ∼59 nm. Similarly, nanoassembly was also noticed during nanoparticles synthesis by *P. zeylanica* root extract where very small spherical nanoparticles flocked together to form larger nanostructures (Salunke et al., 2014). The magnitude of zeta potential is the key determinant of potential stability of colloid and the particles with zeta potential values more positive than +30 mV or more negative than −30 mV are considered to be most stable. In contrast, the colloids are least stable at the isoelectric point, where the zeta potential is zero. Herein, the ζ values varied in the range from −1 to −15 mV, depending upon the type of nanoparticles. The AgPtNHs were more stable compared to PtNPs due to more negative zeta potential. Thus they remained more suspended as a stable colloidal solution unlike the PtNPs that settled faster. AgNPs formed uniform homogenous suspension with maximum negative zeta potential. Saeb et al. (2014) reported synthesis AgNPs using *Escherichia hermannii* (SHE), *Citrobacter sedlakii* (S11P), and *Pseudomonas putida* (S5) where the therapeutic potential was found to be a function of particle size and stability as reflected by its zeta potential. AgNPs synthesized using SHE exhibited the best antimicrobial activity due to small size (4–12 nm) and stability (-22 mV).

The phytochemistry of medicinal plants plays a very significant role in reducing the metal ions to the corresponding nanoparticles as well as their stabilization. The FTIR analysis showed that synthesis and capping of AgPtNHs might be brought about by the functional groups specific to flavonoids, terpenoids, phenanthrenes, amino acids, proteins, and glycosides present within the DBTE extract. Carbonyl stretch from carboxylic acid and C–N stretching vibration of aromatic compounds were also observed. These observation can be strongly rationalized due to the compounds such as diosgenin, dioscorin, dioscin, phytosterols, alkaloids, tannin, starch, ascorbic acid, beta-carotene, protein, riboflavin, and many others which are reported in *D. bulbifera* tubers or rhizomes (Ghosh et al., 2015a; Kundu et al., 2020).

Further, the effect of the phytogenic nanoparticles was checked for their antimicrobial properties. Increased cases of multidrug resistance among bacteria have become a global threat. Microbial pathogens generally gain antibiotic resistance by the following mechanisms: (a) alteration of microbial drug target proteins, (b) enzymatic degradation or inactivation of drug, (c) decreased membrane permeability, and (d) increased efflux of the drug (Kumar et al., 2013). Hence, it is very critical to explore the complementary and alternative therapies to treat microbial infections. Metal nanoparticles with the combined effect of two or more metals can be useful in designing new antimicrobial agents. In this study, the bioreduced AgPtNHs exhibited superior antimicrobial activity against the test pathogenic bacteria which was comparatively higher than the AgNPs and PtNPs, individually.

**FIGURE 8:**
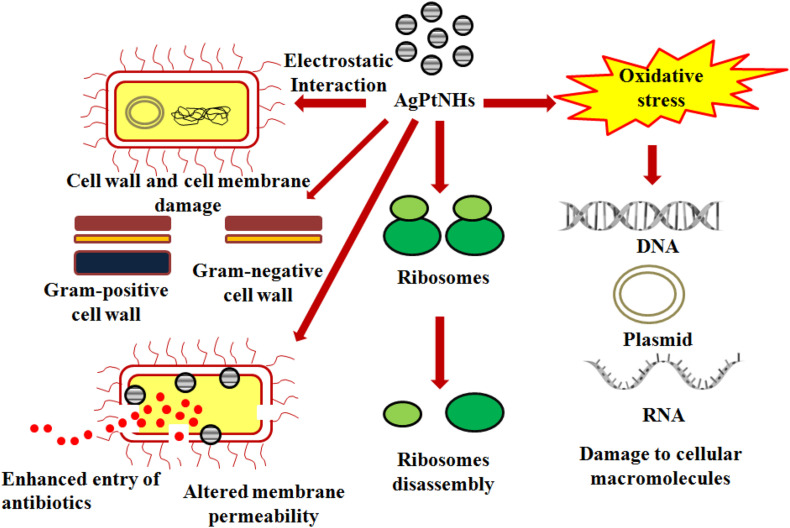
Possible mechanism of antimicrobial synergy of AgPtNHs in combination with antibiotics.

Although there are well established mechanisms on antimicrobial properties of AgNPs, the rationale behind its synergy with the PtNPs are still unknown. AgNPs can depolarize cell membrane in bacteria which alters membrane permeability resulting in leakage of the bacterial metabolites leading to cell death (Vazquez-Muñoz et al., 2019). Likewise incorporation of Pt containing hybrid nanoparticles like Ti-PtNPs resulted in enhanced killing of the bacterial pathogens due to leakage of cytosolic proteins (Selvi et al., 2020). Moreover, combination of Ag and Pt components might have multiple mode of action and cascade of events behind synergistic enhancement of antimicrobial efficiency. The hybrid metal nanoparticles may bind more strongly with the bacterial cell wall and generate oxidative stress due to production of free radical that can include superoxide (O^2–^) and hydroxide radicals (OH). Additionally, binding of the nanoparticles with the thiol group of essential enzymes can lead to their inactivation through the respiratory burst activity. Furthermore, the AgPtNHs might have interacted with nucleic acid (DNA) and interrupted the cellular transport system. The possible mechanism involved in the antibacterial activity of AgPtNHs is displayed in Figure 8. Another reason behind antimicrobial synergy of AgPtNHs in combination with antibiotics might be due to the disruption of the cell wall and membrane, that increase the permeability and facilitates easy entry of antibiotics within the bacterial cells. Hence, the bacteria became more susceptible to the antibiotics in the presence of the AgPtNHs. Also, the simultaneous action of antibiotics and AgPtNHs will make it difficult for pathogenic bacteria to develop resistance. Hence, this combinational therapy can be further developed as novel formulations to treat nosocomial infections.

Raghupathi et al. (2011) reported that antimicrobial activity of the nanoparticles is size dependent. The smaller nanoparticles of size 12 nm showed more potent antimicrobial activity against *S. aureus* compared to the larger particles (100 nm). Hence, the AgPtNHs with the size distribution between 20 and 80 nm probably showed an intermediate activity. However, this hurdle can be overcome by synthesizing similar nanohybrids employing solvothermal synthesis using optimal precursors. Raghupathi et al. (2011) used solvothermal synthesis for fabricating ZnO NPs with size between 2 and 25 nm which were otherwise 100 nm.

In another study, Singh et al. (2018) also reported that small particles interact more easily with the cell surface and are internalized into the cytoplasm due to less spatial hindrance. The greater surface area of smaller nanoparticles can more effectively interact with the cellular components of the bacteria after entry within the cytoplasm. AgNPs can release Ag^+^ ions via oxidation resulting in enhanced generation of reactive oxidative species that damages the cellular components and eventually results in cell death. Hence, fabricating AgPtNHs in future with smaller size will be an additional contributing factor to their enhanced antibacterial effect.

Pathogenic bacteria are highly responsible for nosocomial (hospital-borne) infections, which mainly occur due to biofilm formation on indwelling medical devices and implants such as heart valves, pacemakers, vascular grafts, catheters, prosthetic joints, intrauterine devices, sutures, and contact lenses (Ghosh et al., 2020). Several biofilm driven infections include dental caries and root canal infections, bacterial vaginosis, cardiovascular disease, diabetic foot infections, and urinarytract infections along with prostatitis (Malik et al., 2013; Lanter et al., 2014; Delcaru et al., 2016; Jung et al., 2017; Walsh, 2020). Interestingly, AgPtNHs exhibited higher antibiofilm activity in compared to individual AgNPs or PtNPs. Previous reports showed that Ag nanocomposites (64 μg/mL) and cationic amphiphile could penetrate into the biofilms and eradicated them. Furthermore, AgNPs were reported to penetrate and disperse into biofilm matrixes and then deliver Ag^+^ flux to the bacterial wall to eradicate the biofilms (Dai et al., 2017). Our results showed that Gram-positive bacteria were more resistant to AgPtNHs compared to Gram-negative bacteria. This variation in antimicrobial and antibiofilm activity between Gram-positive and Gram-negative microorganisms is often attributed to difference in the cell wall structures (Al-Sharqi et al., 2019). *S. aureus* may have a stronger defense system against AgPtNHs due to the presence of a thicker cell wall that prevents the action of the AgPtNHs, rendering the bacterium comparatively more resistance to the antimicrobial activity of AgPtNHs. Moreover, the cell wall of Gram-negative bacteria possesses a stronger negative charge than Gram-positive bacteria due to the presence of lipopolysaccharides (LPS), which promotes adhesion of AgPtNHs, causing the bacteria to be more susceptible to AgPtNHs antimicrobial action. Hence, electrostatic attraction between negatively charged bacterial cells and positively charged AgPtNHs is crucial for the bactericidal and antibiofilm efficacy.

The strong antimicrobial synergy and antibiofilm activity suggest that phytogenic hybrid nanoparticles composed of elemental silver and platinum could be valuable in discovering new nanomedicine for treating pathogenic bacterial infections.

## Conclusion

In this work the result showed that the AgPtNHs were synthesized by using aqueous extract of *D. bulbifera* tuber to evaluate their antimicrobial synergy in combination with the antibiotics against both Gram-positive and Gram-negative bacterial pathogens. The nanocluster shaped AgPtNHs were monodispersed with an average diameter of ∼59 nm. Phytochemicals present in DBTE facilitated the synthesis of AgPtNHs by reducing the metal ions and also their stabilization. Three test pathogens, *E. coli*, *P. aeruginosa*, and *S. aureus* were inhibited by AgPtNHs alone while the combination of AgPtNHs with antibiotics such as rifampicin and novobiocin showed high antimicrobial synergy. Biofilm formation was significantly inhibited by the phytogenic AgPtNHs which irreversibly eradicated bacterial biofilms on glass surfaces. The obtained nanocomposites could effectively eradicate bacterial biofilm at a low concentration of 10 μg/well. Combined treatment of AgPtNHs and antibiotics for killing bacteria is advantageous as it would lower the concentration of antibiotics used which otherwise triggers multidrug resistance. Thus, combined antimicrobial therapy is expected to be more efficient for preventing bacterial regrowth than conventional antibacterial agents.

## Data Availability Statement

The original contributions presented in the study are included in the article/supplementary material, further inquiries can be directed to the corresponding author/s.

## Author Contributions

BR, GS, SK, KB, and SS performed all the experiments, analyzed the data, and interpreted the results and wrote the initial draft of the manuscript. NK supervised and designed the imaging experiments and analyzed the data, and wrote the manuscript. PK supervised the characterization experiments, analyzed the data, and participated in the writing of the manuscript. SG conceived the idea, designed and supervised the study, analyzed the data, and wrote the manuscript. PK, SG, and NK revised and finalized the manuscript. All authors contributed to the article and approved the submitted version.

## Conflict of Interest

The authors declare that the research was conducted in the absence of any commercial or financial relationships that could be construed as a potential conflict of interest.
